# Characterisation of extracellular vesicles isolated from hydatid cyst fluid and evaluation of immunomodulatory effects on human monocytes

**DOI:** 10.1111/jcmm.17894

**Published:** 2023-08-02

**Authors:** Mojdeh Khosravi, Hanieh Mohammad Rahimi, Abdoreza Nazari, Kaveh Baghaei, Hamid Asadzadeh Aghdaei, Shabnam Shahrokh, Meysam Sharifdini, Ana Claudia Torrecilhas, Fatemeh Mehryab, Hamed Mirjalali, Faezeh Shekari, Mohammad Reza Zali

**Affiliations:** ^1^ Department of Pharmacy and Pharmaceutical Technology and Parasitology University of Valencia Valencia Spain; ^2^ Foodborne and Waterborne Diseases Research Center, Research Institute for Gastroenterology and Liver Diseases Shahid Beheshti University of Medical Sciences Tehran Iran; ^3^ Department of Molecular Systems Biology at Cell Science Research Center Royan Institute for Stem Cell Biology and Technology Tehran Iran; ^4^ Basic and Molecular Epidemiology of Gastrointestinal Disorders Research Center, Research Institute for Gastroenterology and Liver Diseases Shahid Beheshti University of Medical Sciences Tehran Iran; ^5^ Gastroenterology and Liver Diseases Research Center, Research Institute for Gastroenterology and Liver Diseases Shahid Beheshti University of Medical Sciences Tehran Iran; ^6^ Department of Medical Parasitology and Mycology, School of Medicine Guilan University of Medical Sciences Rasht Iran; ^7^ Laboratório de Imunologia Celular e Bioquímica de Fungos e Protozoários, Departamento de Ciências Farmacêuticas Universidade Federal de São Paulo (UNIFESP) Diadema Brazil; ^8^ Department of Pharmaceutics and Pharmaceutical Nanotechnology, School of Pharmacy Shahid Beheshti University of Medical Sciences Tehran Iran

**Keywords:** *Echinococcus granulosus*, extracellular vesicles, human monocyte cell line, hydatid cyst, inflammation

## Abstract

Hydatidosis is a disease caused by the larval stage of *Echinococcus granulosus*, which involves several organs of intermediate hosts. Evidence suggests a communication between hydatid cyst (HC) and hosts via extracellular vesicles. However, a little is known about the communication between EVs derived from HC fluid (HCF) and host cells. In the current study, EVs were isolated using differential centrifugation from sheep HCF and characterized by western blot, electron microscope and size distribution analysis. The uptake of EVs by human monocyte cell line (THP‐1) was evaluated. The effects of EVs on the expression levels of pro‐ and anti‐inflammatory cytokines were investigated using quantitative real‐time PCR (RT‐PCR), 3 and 24 h after incubation. Moreover, the cytokine level of IL‐10 was evaluated in supernatant of THP‐1 cell line at 3 and 24 h. EVs were successfully isolated and showed spherical shape with size distribution at 130.6 nm. After 3 h, the expression levels of pro‐inflammatory cytokine genes (*IL1Β*, *IL15* and *IL8*) were upregulated, while after 24 h, the expression levels of pro‐inflammatory cytokines were decreased and *IL13* gene expression showed upregulation. A statistically significant increase was seen in the levels of IL‐10 after 24 h. The main mechanism of the communication between EVs derived from HCF and their host remains unclear; however, time‐dependent anti‐inflammatory effects in our study suggest that HC may modulate the immune responses via EVs.

## 1 INTRODUCTION

Human cystic echinococcosis (Hydatidosis, or hydatid disease) is a zoonotic neglected disease (ZND) caused by the larval stage of *Echinococcus granulosus*, which is considered as a public health concern, particularly in regions with livestock.^1^, ^2^ *E. granulosus* sensu stricto, *E. equinus*, *E. ortleppi* and *E. canadensis* cause cystic echinococcosis (CE), while *E. vogeli* and *E. oligarthra* are causative agents for polycystic echinococcosis, and *E. multilocularis* causes alveolar echinococcosis, which could be lethal.^1^, ^2^

Hydatidosis has a worldwide distribution with reports from all continents. The prevalence of this disease can reach 5%–10% in certain endemic regions; however, the surgical incidence rate of human CE in a global scale seems to be less than 10/100000.^3^ In Nepal, a less developed country, CE (251 disability‐adjusted life year [DALYs] [95% CrI: 105–458]) is the third zoonotic parasite from 2000 to 2012,^4^ while in a similar time period in Italy, a developed country, the annual DALYs for CE during 2001–2014 was estimated to be 223.35.^5^ However, the latest global estimation suggests 1–3 million DALYs per year for CE.^3^

Hydatidosis occurs due to the metacestode stage of *E. granulosus*, which involves almost all organs.^6^ The hydatid cyst (HC) structure from the outer to the inner layers consists of a fibrotic layer, which is resulted from host immune responses, laminated layer (LL), germinal layer (GL), which is the origin of brood capsule, and HCs fluid (HCF).^2^, ^6^ It was well‐documented that HCF is a heterogenic fluid containing several proteins, which are originated from protoscoleces, GL, and even host.^7^ Excretory/secretory (E/S) materials, produced by protoscoleces, may permeate across LL and fibrotic layer, and manipulate host immune responses.^8^, ^9^ Therefore, it seems that there is a cross‐talk between metacestode stage of *E. granulosus* and its hosts.^9^

Extracellular vesicles (EVs) are released from different cell types and microorganisms. EVs play crucial role in cell signalling, modulating of the immune responses, and communication between host cells and infectious agents.^10^, ^11^, ^12^, ^13^, ^14^ Based on the biogenesis, EVs have been categorized as exosomes, microvesicles and apoptotic bodies.^15^, ^16^ EVs mostly carry a complex of components and show features of their mother cell. EVs contain lipids, proteins, genetic materials (DNA, RNA and micro RNAs) and metabolites.^17^, ^18^, ^19^, ^20^ During recent decades and alongside a plenty of studies on characterisation and applications of EVs, many studies have been conducted on the isolation, characterisation and biomedical applications of EVs, which are excreted from parasites.^14^, ^21^ A body of evidence have indicated an important role for EVs in pathogenesis of parasites via either carrying virulence factors or modulation of the host immune responses.^11^, ^13^, ^22^, ^23^, ^24^

The modulatory effects of EVs, derived from human and sheep HCF, were demonstrated on murine PBMC and T cells, and sheep PBMC.^25^, ^26^ However, a lot of subjects about host–parasite communication through EVs remain unclear. In the current study, EVs from fertile sheep HCF were isolated and time‐dependent effects of EVs on inflammasome NLRP3 and pro/anti‐inflammatory cytokines were investigated. In addition, uptake of EVs by human monocyte cell line (THP‐1) were evaluated.

## 2 MATERIALS AND METHODS

### 2.1 Ethics approval and consent to participate

All experimental protocols were approved by the Research Institute for Gastroenterology and Liver Diseases, and all procedures performed in this study were approved by the ethical standards (IR.SBMU.RCGLD.REC.1398.015) released by Ethical Review Committee of the Research Institute for Gastroenterology and Liver Diseases, Shahid Beheshti University of Medical Sciences, Tehran, Iran. In addition, all methods were carried out in accordance with relevant guidelines and regulations, and all authors complied with the ARRIVE guidelines.

### 2.2 Parasite preparation

To obtain HCF, a liver full of fertile HCs from a sheep, which was slaughtered in an abattoir located in Guilan province, north of Iran, was aseptically transferred to the Foodborne and Waterborne Diseases Research Center of the Research Institute for Gastroenterology and Liver Diseases, Shahid Beheshti University of Medical Sciences, Tehran, Iran. In the parasitology lab, all morphologic and parasitology features of cysts were recorded, the HCF was aseptically aspirated from cysts and transferred to sterile 50‐mL conical centrifuge tubes. To confirm the fertility of cysts, 10 μL of HCF was investigated by optical microscope and the number of protoscoleces were counted by vital staining (trypan blue). The genotype of *E. granulosus* sensu *lato* was characterized based on the amplification and sequence analysis of identical fragment of the cytochrome oxidase (*COX*) 1 gene in the Guilan University of Medical Sciences.^27^

### 2.3 EVs isolation and characterisation

To isolate EVs from HCF, differential centrifugation was employed on 500 mL of fresh HCF to separate EVs. Isolated EVs were analysed regarding surface markers, shape and size, based on MISEV2018 guideline.^28^ Briefly, HCF was centrifuged at 300 × ***g*** for 15 min, and supernatant was transferred to sterile 50‐mL conical centrifuge tubes for further analyses. Collected supernatant was centrifuged at 3000 × ***g*** for 10 min to remove the cell debris. The large vesicles were isolated by high‐speed centrifugation at 20,000 × ***g*** for 30 min at 4°C. To collect the small EVs, ultracentrifugation at 100,000 × ***g*** for 120 min at 4°C in a 45Ti rotor (Beckman Coulter, Inc.) was performed. The resultant pellet was then re‐suspended in PBS and spun again at 100,000 × ***g*** for 120 min at 4°C. Following the last wash, the EVs were re‐suspended in PBS and stored at −80°C. The ultracentrifugation and all characterising processes were carried out in the Department of Stem Cells and Developmental Biology at Royan Institute for Stem Cell Biology and Technology.

### 2.4 DLS analysis

The suspension of EVs in PBS was loaded into a cuvette. Particle size distribution of the EVs was measured via dynamic light scattering (DLS, Zetasizer nano range). Fluctuations of light scattering intensity were evaluated using a laser to calculate the size of EVs. Data were analysed using Malvern Zetasizer Software (Malvern Instruments, v7.03).

### 2.5 Western blot

The presence of EV markers (CD63 and CD81) was checked in both parasite lysate and EVs. Protein was extracted from protoscoleces of HC by repeated freeze thawing followed by a sonication in SDS‐PAGE sample buffer, as mentioned previously.^29^ To extract total protein, EVs were boiled in SDS‐PAGE sample buffer followed by sonication for 5 min. BCA assay was employed to estimate the protein content of samples. In addition, the origin of EVs was investigated using analysis of the interaction between HC‐positive human serum and EVs via western blot analysis.

To perform western blot, 20 μg (based on BCA assay of protein content) of EVs sample was mixed with SDS‐PAGE loading buffer and sonicated for 5 min followed by heating at 95°C for 5 min. The sample was then loaded onto 10% SDS‐PAGE gel. Samples were transferred onto polyvinyl difluoride (PVDF) membrane (Millipore), and the membrane was blocked with the solved bovine serum albumin (5%) (BSA) in Tris‐buffered saline (TBST) special (Tween 20: 0.1%). The CD63 (1:500, Abcam: Ab8219) and CD81 (1:500, Santa Cruz: SC7637), as positive markers, and Calnexin (1:500, Santacruz: Sc11397), as a negative marker, were incubated overnight with the membrane. Then, the membrane was washed by TBST special (1X) and incubated with a secondary antibody (1:50000, Sigma) for 1 h at room temperature. After washing the membrane with TBST special (1X), the enhanced chemiluminescence (ECL) solution was added. Then, the protein bands were detected by a chemiluminescence device (Uvitec). All western blot analyses were performed in two repeats and results were same.

### 2.6 Electron microscopy

The morphology of EVs was evaluated by a scanning electron microscope (SEM). 150 ng suspension of EVs was dried on a glass slide, coated by gold (SCD 005, Bal‐Tec), and analysed by the SEM (XL30, Philips).

### 2.7 EV staining and uptake analysis

In order to reduce the false‐positive signal that is common for lipophilic dyes, Calcein labelling was employed. To prepare a 10X solution (1 mM), the Calcein‐AM (1 mg; Invitrogen: C3099) was solubilized in DMSO (1 mL). This stock was diluted to a final concentration of 1X as a working solution. The EV sample (20–100 μL, 40–200 μg) was added to 100 μL of calcein‐AM working solution. The surplus Calcein‐AM was removed using exosome spin columns (MW 3000; Invitrogen: 4484449). Upon the hydration of the column with 650 μL of RNase‐free water or PBS, the column was tapped. Spin column was placed in a 2‐mL collection tube and centrifuged at 750 × ***g*** for 2 min at room temperature. The EV sample (equal to 40–200 μg) was directly added to the top of the column and centrifuged at 750 × ***g*** for 2 min at 4°C. The EVs samples were eluted into the elution tube and incubated at 37°C for 20 min.^30^ Afterwards, labelled EV‐suspension was incubated with THP‐1 cells, which were cultivated in FBS‐free cell medium in a T25 cell culture flask for 4 h at 37°C. Cells were washed with PBS, fixed in 4% paraformaldehyde (PFA), and imaged using a common FITC channel (calcein excitation max 495 nm/emission max 516 nm) in fluorescence microscopy (Carl Zeiss Microscopy).

### 2.8 THP‐1 cell line culture, differentiation to human macrophages

The THP‐1 cell line (ATCC TIB‐202) was maintained in Roswell Park Memorial Institute (RPMI) 1640 medium (Gibco, Thermo Fisher Scientific) with penicillin–streptomycin (1% penicillin/streptomycin), supplemented by 10% heat‐inactivated FBS. After cultivation of THP‐1 cells in cell culture flasks, 1 × 10^6^ of cells were transferred to flat‐bottom 6‐well adherent cell culture plate, and were incubated at 37°C in 5% CO_2_. The cells were then treated with 30 ng/mL of phorbol 12‐myristate 13‐acetate (PMA; Santa Cruz Biotechnology Cat No. sc‐3576) for 48 h to differentiate THP‐1 monocytes into M0 macrophages. To confirm differentiation, all THP‐1 cells were morphologically screened. Afterward, supernatant was removed and PMA‐ and LPS ‐free cell culture medium (RPMI 1640 medium with 10% FBS and 1% antibiotic) was added. The cells were rested for 24 h prior to experiments.^31^ Finally, supernatant was discarded, cells were washed twice with sterile PBS (pH = 7–8), EV‐free RPMI 1640 medium without FBS was added to plates, and the cells were treated with 100 μg/mL of isolated EVs. Treatment with 20 ng/mL LPS (derived from *E. coli* O111:B4; Santa Cruz Biotechnology Cat No. sc‐3535) was performed to compare induction patterns. A well full of PMA‐activated THP‐1 cell without any treatment, neither by HCF EVs nor LPS, was considered as control group. All groups were duplicated and evaluated 3 and 24 h after exposure.

### 2.9 RNA isolation, complementary DNA (cDNA) synthesis and quantitative real‐time PCR

Total RNA was extracted after 3 and 24 h by total RNA extraction kit (Yekta Tajhiz Azma), according to the manufacturer's protocol. DNase (Thermo Fisher Scientific™) treatment was performed to remove the probable residual DNA and to improve the quality of the extracted RNA, according to the manufacturer's protocol. The RNA solution was finally collected and stored at −70 ° C until cDNA synthesis. Prior to cDNA synthesis, the concentration of purified RNA samples was determined by NanoDrop (NanoDrop Technologies) apparatus and RNA was adjusted (normalized). cDNA was constructed using cDNA synthesis kit (Yekta Tajhiz Azma), according to the manufacturer's protocol. To study the expression levels of the *IL1Β*, *NLRP3*, *IL8*, *IL15*, *TNFΑ*, *IFNG*, *IL4*, *IL13* and *TGFΒ* genes, qRT‐PCR using specific primers was employed (Table 1). Real‐time PCR was performed using Rotor‐Gene Q (Qiagen, Germany) thermocycler in a 20 reaction mixture containing 10 μL SYBR Green qPCR master mix 2X (Ampliqon), 5 μM of primers, and template cDNA, based on MIQE guideline: cycling reactions starting with one cycle of 50°C (2 min) and 95°C (1 min), followed by 40 cycles at 95°C (15 s) and 60°C (1 min). Melting curves were determined with ramping from 75°C to 90°C s^−1^, as previously described.^31^ The relative expression of targeted genes compared to the housekeeping gene was calculated using 2 ^(‐ΔΔCt)^ method. All qRT‐PCR procedures were performed following the MIQE guideline. Each reaction was performed in duplicate, and the *ACTΒ* gene was considered as housekeeping gene.

**TABLE 1:** Oligonucleotides used for amplification of each target gene.

Genes	Sense primer(5′‐3′)	Antisense primer(5′‐3′)	Length (bp)	Refs
*IL1Β*	CAGGGACAGGATATGGAGCAAC	CATCTTTCAACACGCAGGACAG	133	^32^
*NLRP3*	AAGGAAGTGGACTGCGAGA	TCAAACGACTCCCTGGAAC	127	^33^
*IL8*	TGGCTCTCTTGGCAGCCTTC	TGCACCCAGTTTTCCTTGGG	238	^34^
*IL15*	TGTCTTCATTTTGGGCTGTTTCA	GAATACTTGCATCTCCGGACTC	227	^35^
*TNFΑ*	AGCCCATGTTGTAGCAAACC	TGAGGTACAGGCCCTCTGAT	134	^36^
*IFNG*	TGACCAGAGCATCCAAAAGA	CTCTTCGACCTCGAAACAGC	230	^37^
*IL4*	CGAGTTGACCGTAACAGACAT	CGTCTTTAGCCTTTCCAAGAAG	280	^38^
*IL13*	CAGAACCAGAAGGCTCCGCTC	CGGACATGCAAGCTGGAAAACTG	194	This study
*TGFΒ*	ATGCCCGTATTTATGGAGTT	ATTGTCATTTTGGTCTTGCC	234	^39^
*BACT*	ATGTGGCCGAGGACTTTGATT	AGTGGGGTGGCTTTTAGGATG	107	^40^

Abbreviations: AIM, absent in melanoma; BACT, beta actin; IL, interleukin; NLRC, nod‐like receptor CARD domain containing; NLRP, nod‐like receptor pyrin domain containing.

### 2.10 Cytokine assay by ELISA

Culture supernatants were collected after the treatments and stored at −80°C for cytokine analysis. The secretion level of IL‐10, an important anti‐inflammatory cytokine, was measured with standard enzyme immunoassays (ELISA) kits, according to the manufacturer's protocols. Raw data were analysed by the web software MyAssays (https://myassays.com/assay.aspx?id=787), and the concentrations were calculated by a Four Parameter Logistic (4PL) curve.

### 2.11 Statistical analysis

Student's *t*‐test was applied to analyse relative gene expressions at different time points in comparison to the housekeeping gene. *p* < 0.05 was considered statistically significant. Statistical analysis was performed using GraphPad Prism software version 8.3.0.538.

## 3 RESULTS

### 3.1 Parasitology

The HCF was aspirated from liver of a HC‐infected sheep. The size of HCs was between 3 and 15 cm and there was no evidence of calcification. The HCF contained protoscoleces, daughter cysts and LL (Figure S1). The genotype of isolated *E. granulosus* sensu *lato* was G1.^27^

### 3.2 EVs characterisation and uptake

**FIGURE 1:**
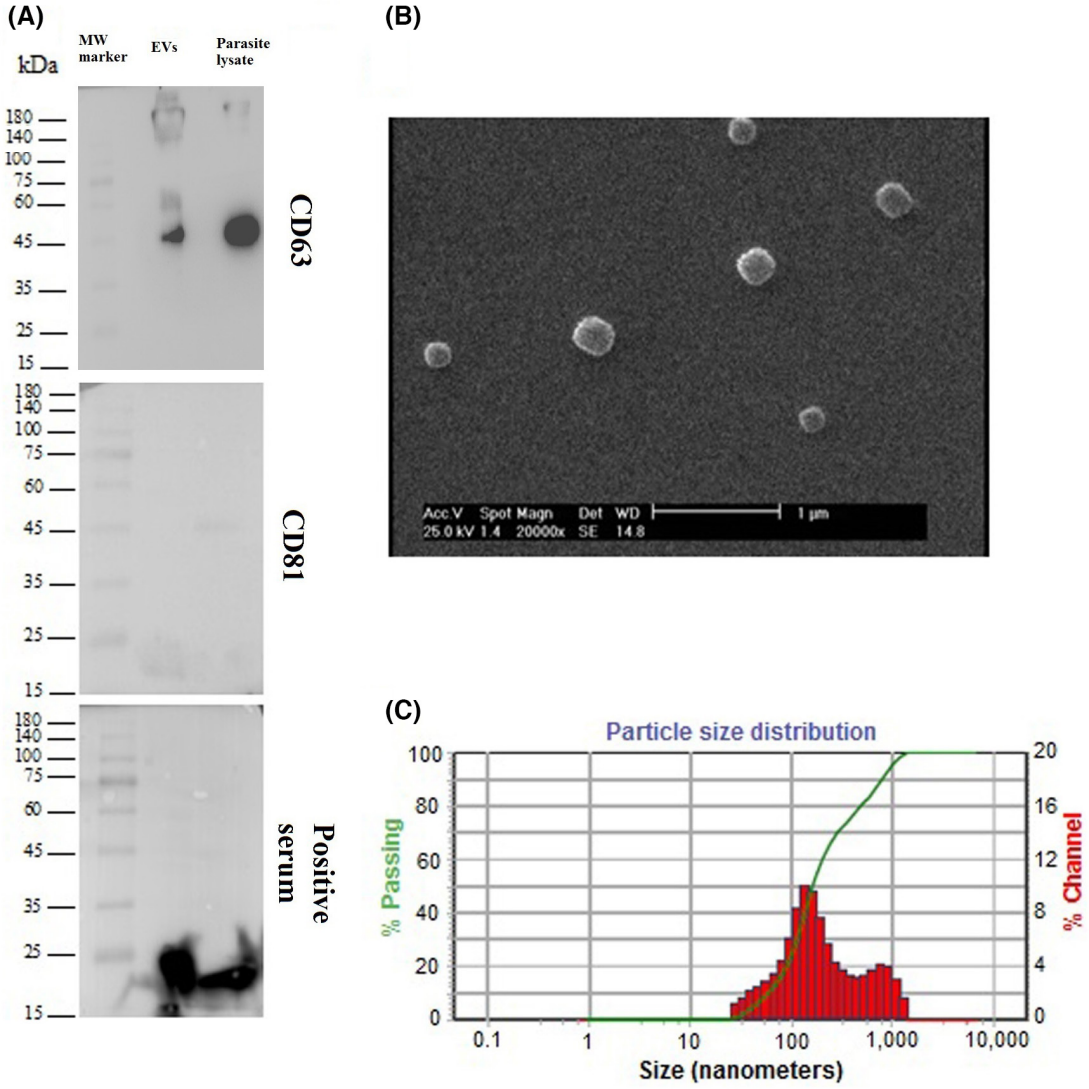
The immunologic and morphometric analyses of isolated EVs and an immunologic comparison between EVs and the parasite lysate. (A) The westernblot analysis suggests the presence of CD63 marker and the lack of CD81 marker. In addition, EVs and parasite lysate exhibited similar expression of specific markers. (B) The analysis of SEM microscope represented spherical shape EVs isolated from HCF. (C) DLS analysis shows that EVs mostly presented size at 130.6 nm.

Western blot analyses confirmed the expression of CD63 marker, while Calnexin and CD81 were absent in EVs samples (Figure 1A, Figure S2). Our findings confirmed the presence of CD63 and absence of CD81 in protein lysate of protoscoleces, as well (Figure 1A). The SEM and morphology revealed round shape vesicles (Figure 1B). The DLS analysis showed average size distribution, in which most of EVs (~ 74%) represented a peak at 130.6 nm diameter (Figure 1C). HC‐positive human serum showed an interaction with protein lysate of protoscoleces and EVs (Figure 1A, Figures S3 and S4). THP‐1 cells were incubated with EVs, which were labelled by Calcein‐AM that the results showed an internalization rate over 60% and bright fluorescent signals at 4 h after incubation (Figure S5).

### 3.3 Relative gene expression of qReal‐time PCR panel

**FIGURE 2:**
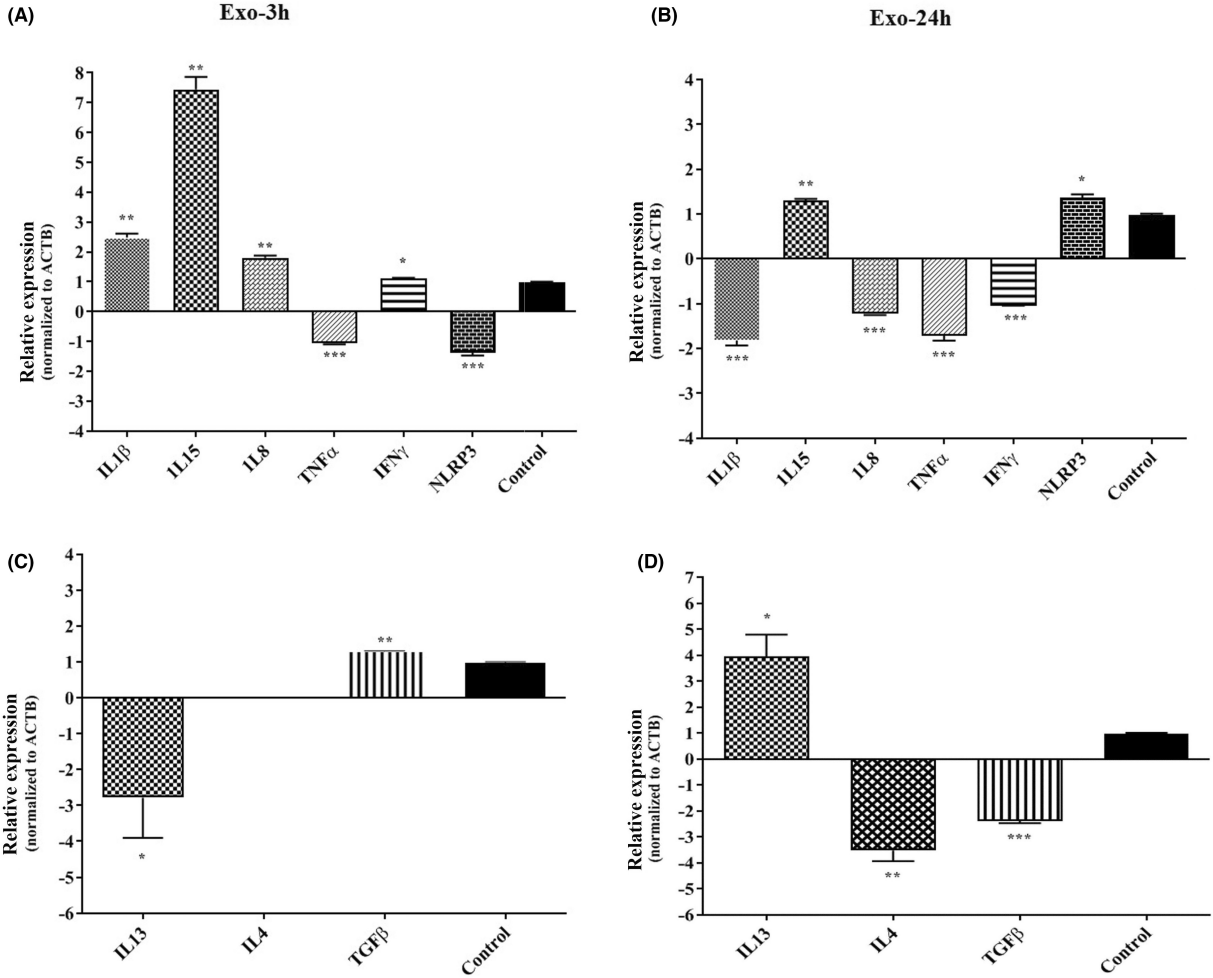
The comparison of the expression levels of inflammatory *IL1Β, IL15, IL8, TNFΑ, IFNG* and *NLRP3* genes in THP‐1 cell line co‐incubated with HCF derived EVs, compared to the housekeeping gene (*ACTB*) for (A) 3 h and (B) 24 h. This picture shows also the expression of anti‐inflammatory biomarkers *IL4, TGFΒ*, and *IL13* genes in THP‐1 cell line co‐incubated with HCF derived EVs (C) 3 h and (D) 24 h after exposure. The expression levels of pro‐inflammatory cytokines are time‐dependently decreased 24 h after exposure to HCF EVs. * *p* < 0.05; ** *p* < 0.01; *** *p* < 0.001. ACTB, Beta Actin; C, control; IFN: interferon; IL, Interleukin; NLRP, nod‐like receptor pyrin domain containing; TGF, tumour growth factor; TNF, tumour necrosis factor.

The relative gene expression analysis of inflammatory biomarkers showed statistically significant upregulation of *IL1Β*, *IL15*, *IL8* and *IFNG* after 3 h, while *TNFΑ* and *NLRP3* showed statistically significant downregulation (Figure 2A). The anti‐inflammatory biomarkers, *IL13*, *TGFΒ* and *IL4*, were significantly downregulated, upregulated and suppressed, respectively (Figure 2B). Almost all the tested genes showed downregulation after 24 h except *NLRP3* and *IL15* (Figure 2C). In addition, the expression changes of anti‐inflammatory biomarkers were statistically significant (*p* = 0.0003), while only *IL13* showed upregulation (Figure 2D).

**FIGURE 3:**
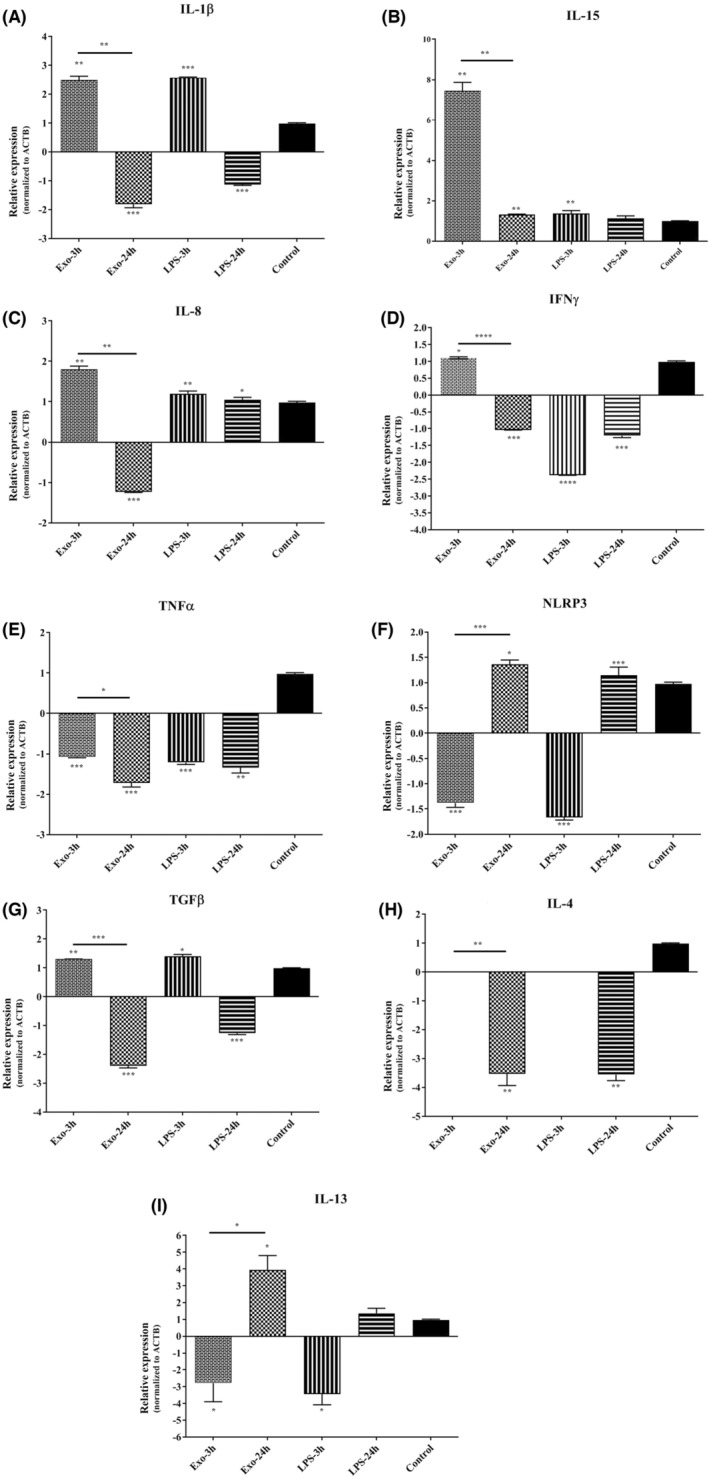
The expression levels of (A) *IL1Β*, (B) *IL15*, (C) *IL8*, (D) *IFNG*, (E) *TNFΑ*, (F) *NLRP3*, (G) *TGFΒ*, (H) *IL4*, and (I) *IL13* genes in THP‐1 cell line co‐incubated with HCF derived EVs, compared to the housekeeping gene (*ACTB*), 3 and 24 h after exposure. This picture shows that the expression levels of pro‐inflammatory cytokines are time‐dependently decreased 24 h after exposure to HCF derived EVs. * *p* < 0.05; ** *p* < 0.01; *** *p* < 0.001; **** *p* < 0.0001. ACTB, Beta Actin; C, Control; IFN: Interferon; IL, Interleukin; NLRP, Nod‐like receptor pyrin domain containing; TGF, Tumour growth factor; TNF, Tumour necrosis factor.

The expression of *IL1Β*, compared to the *ACTB* was increased after 3 h, while it was significantly reduced 24 h after co‐incubation with HCF EVs. The comparison between time points showed statistically significant difference (*p* = 0.0008) (Figure 3A). Compared to the housekeeping gene, the highest upregulation during 3 h after co‐cultivation with HCF EVs was seen in the expression of *IL15*, while it was dramatically decreased after 24 h (Figure 3B). The expression level of *IL8* gene was significantly upregulated and downregulated 3 h and 24 h after the exposure, respectively. (Figure 3C). The changes of *IFNG* was not statistically significant at 3 h, but showed downregulation at 24 h. (Figure 3D). The expression of *TNFΑ* was significantly decreased at 3 and 24 h after the exposure (Figure 3E). In addition, the expression of *NLRP3*, which plays crucial role in innate immunity and the production/ secretion of IL‐1β, was significantly decreased at 3 h, while it showed significant overexpression after 24 h (Figure 3F).

The expression of *TGFΒ* was significantly increased after 3 h, but it was significantly downregulated at 24 h (Figure 3G). Compared to the housekeeping gene, the relative gene expression of *IL4* was suppressed in 3 h after the exposure, while it was significantly decreased during 24 h after co‐culture (Figure 3H). Moreover, the *IL13* gene was downregulated at 3 h after the exposure, while its expression was increased after 24 h (*p* = 0.038) (Figure 3I). All expression changes of studied factors and their significance values are summarized (Table 2).

**TABLE 2:** The relative expression of investigated factors, compared to the *ACTB* housekeeping gene, in THP‐1 cell line treated by EVs isolated from HC using real‐time PCR.

Factors	3 h		24 h		Statistical comparisons between time points (*p‐*values)
	The mean of relative expression ± SD	*p‐*values	The mean of relative expression ± SD	*p‐*values	
*IL1B*	2.487 ± 0.133	0.0041	−1.818 ± 0.117	0.001	0.008
*IL15*	7.44 ± 0.427	0.0022	1.304 ± 0.036	0.0078	0.0024
*IL8*	1.8 ± 0.078	0.0054	−1.23 ± 0.021	0.0002	0.004
*IFNG*	1.106 ± 0.027	0.053	−1.04 ± 0.002	0.0002	< 0.0001
*TNFA*	−1.068 ± 0.029	0.0003	−1.718 ± 0.104	0.0008	0.0136
NLRP3	−1.382^a^ ± 0.085	0.0008	1.362 ± 0.083	0.026	0.0009
*TGFB*	1.3 ± 0.009	0.0015	−2.395 ± 0.077	0.0003	0.0002
IL‐4	‐^b^		−3.526 ± 0.041	0.0041	0.0067
*IL13*	−2.781 ± 1.117	0.041	3.942 ± 0.852	0.039	0.021

^a^ Negative values indicate downregulation

^b^ This factor was suppressed at 3 h investigation.

Abbreviations: IL, interleukin; INF, interferon; NLRP, nod‐like receptor pyrin domain containing; TGF, tumour growth factor; TNF, tumour necrosis factor.

### 3.4 IL‐10 detection in the supernatant in THP‐1 cell line

**FIGURE 4:**
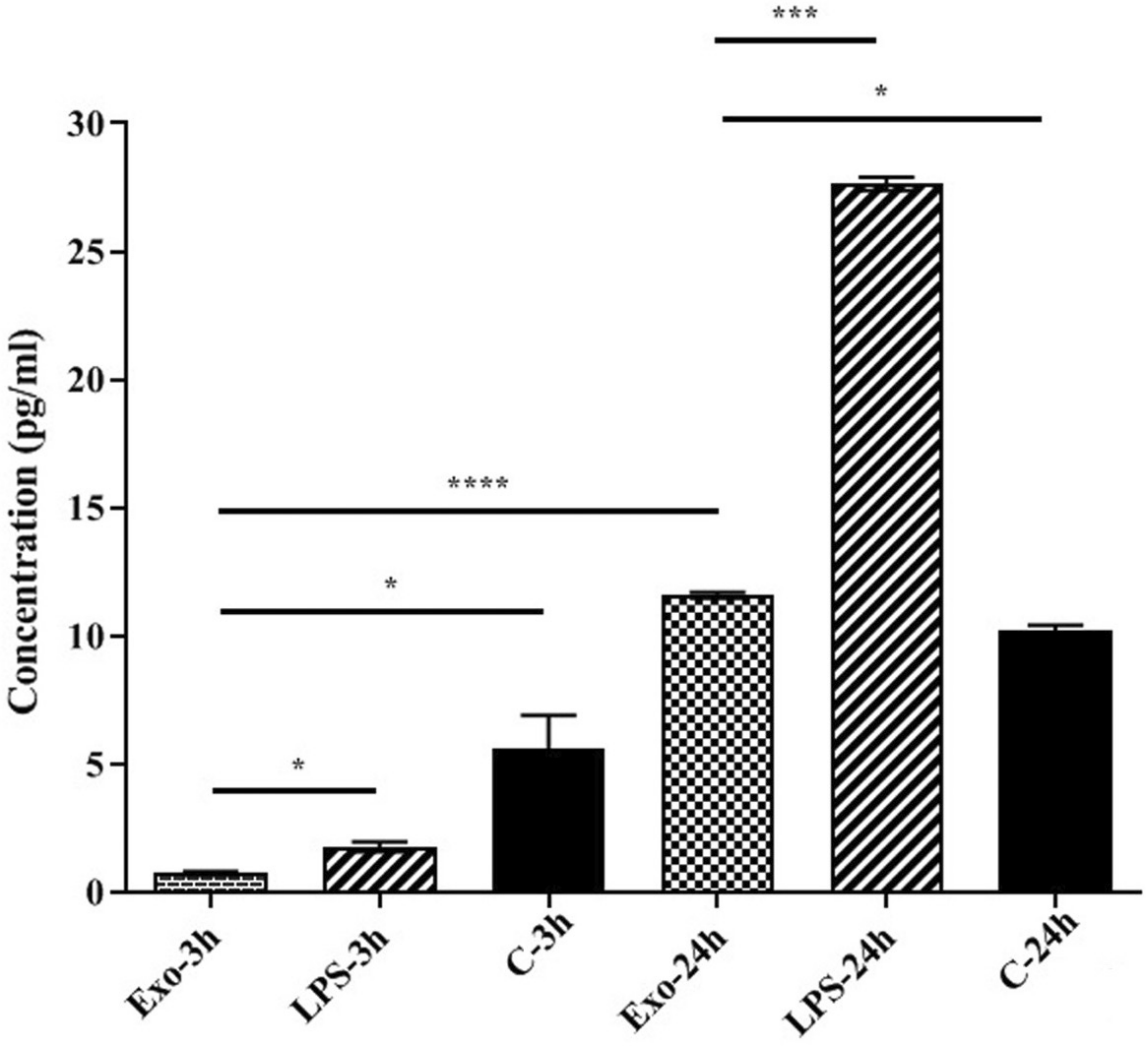
The cytokine release of IL‐10 shows the concentration of this cytokine in 3 h and 24 h after treatment with HCF derived EVs. It seems that treatment with HCF EVs decreases the released concentration of IL‐10. * *p* < 0.05; *** *p* < 0.001; **** *p* < 0.0001. IL, interleukin.

The level of IL‐10 in the cell culture supernatant was evaluated and the results showed low secretion of the cytokine at 3 h compared to control (0.716 pg/mL in EVs‐3 h vs. 5.596 pg/mL in control; *p* = 0.033). The cytokine release in 24 h showed statistically significant increase (*p* <0.0001) compared to control (11.59 pg/mL in EVs‐24 h vs. 10.18 pg/mL in control; *p* = 0.0157) (Figure 4).

## 4 DISCUSSION

EVs contain several molecules that play crucial role in infections caused by parasites. These particles modulate and affect the host immune responses.^22^ In the current study, EVs were isolated from HCF using differential centrifugation, which is known as the most common EVs isolation method,^28^, ^41^ and their immunological effects were evaluated on THP‐1.

Although the presence of CD63 was reported from HC, there are conflicting results. For example, Nicolao et al.^42^ suggested the lack of CD63 in HC, while the expression of CD63 in EVs derived from HCF was confirmed based proteome profiling^25^ and transcriptomic assays.^43^ However, our findings revealed the expression of CD63 in EVs. In addition, we showed a cross reactivity of anti‐human CD63 with CD63 in EVs isolated from HC. We could not detect CD81 by anti‐human antibody. The reason for this finding could be the lack of cross reactivity of the anti‐human antibody with parasite antigen or the lack of expression of CD81 in HC.

During the biogenesis of EVs, the endosomal sorting complex required for transport (ESCRT) is one of the most important component in mammalian cells.^44^, ^45^ ESCRT plays important role in packaging and invagination of exosomes.^46^ The presence of ESCRT‐dependent components in helminth‐derived EVs from parasites such as *F. hepatica*,^47^ HCF^48^ and *Taenia pisiformis* ^49^ have been reported. As a result, CD63 was identified in western blot analysis of EVs isolated from HCF. The CD63 is a ESCRT‐independent component that seems to contribute in formation of microvesicular bodies (MVBs) together with ESCRT‐dependent components.^47^, ^50^ The existence of CD63 in the current study suggests not only the presence of ESCRT‐dependent pathways, but also the crucial role of ESCRT‐independent components in the biogenesis of EVs in HC. However, since the impairment in the ESCRT‐dependent^51^ and ‐independent (CD63)^52^, ^53^ pathways critically affects the formation and number of EVs, these components could be considered as potential targets for next generation drugs.^50^

As findings, EVs derived from HCF amplified the transcriptional levels of inflammatory markers during 3 h. Nevertheless, the expression of pro‐inflammatory and immunomodulatory cytokines was downregulated and upregulated, respectively, after 24 h. Although during recent years, focuses on the communication between parasites and host cells via EVs have been increased, the number of studies on the EVs derived from parasites is not too much.^12^, ^24^ EVs can deliver virulence factors of their mother cell to other cells.^18^, ^22^ In this regard, Marshall et al.,^54^ documented the presence of major surface protease (MSP), also known as GP63, in exosomes, which were derived from promastigotes of *Leishmania infantum*. Parasites are able to manipulate the cell cycle via the genetic content of their EVs, as well. Therefore, it seems that EVs derived from parasites play an important role in providing a favour environment for development of parasite through two main pathways: (1) modulation of the immune responses, and (2) inducing transcriptional changes in host cell, to affect the severity of infection.^22^ For example, Liu et al.,^55^ demonstrated the presence of miR‐125 and a helminth‐specific micro RNA (*bantam*) in EVs derived from *Schistosoma japonicum*. They showed that the EVs can provoke murine macrophages, RAW264.7, to increase the proliferation of the cells and upregulate the expression of TNF‐α. The shifting of immune responses from Th1 to Th2 was observed in C57BL/6 mice and bone marrow‐derived macrophages (BMDMs) treated by EVs from *Trypanosoma cruzi*,^56^ and BALB/c mice and mice BMDMs sensed by *L. amazonensis‐*isolated EVs.^57^ This immune response shifting seems to be an effective mechanism, which is employed by protozoa to obscure the immune system, increase the chance of development of protozoa, and increase the parasite burden in host cells. However, the EVs derived from protozoa mostly provoke Th1 immune responses in macrophage RAW264.7 cell line and chickens treated by *Toxoplasma gondii*‐ and *Eimeria tenella*‐derived EVs, respectively.^58^, ^59^ Unlike protozoa, helminths mostly modulate the immune responses by inciting the secretion of anti‐inflammatory chemokines such as IL‐4, IL‐5, IL‐10, IL‐13, and TGF‐β in mice models.^60^, ^61^ Roig et al.^62^ showed that EVs extracted from *F. hepatica* could decrease the levels of pro‐inflammatory cytokines such IL‐6, TNF‐α and IL‐17R in mice that suffered from DSS‐induced colitis. These results were repeated in another study that evaluated the anti‐inflammatory effects of EVs isolated from *Nippostrongylus brasiliensis* on tissue inflammatory cytokines (IL‐6, IL‐1β, IFN‐γ and IL‐17a) in animal model.^60^ These studies together with a couple of other investigations^61^, ^63^ are in concordance with our finding that showed downregulation of pro‐inflammatory chemokines in THP‐1 cells, 24 h after treating with HCF EVs.

As results, EVs isolated from HCF affected the expression levels of pro‐ and anti‐inflammatory chemokines in a time‐dependent manner. During the first 3 h after exposure, pro‐inflammatory cytokines (IL‐1β, IL‐8, IL‐15 and IFN‐γ) were upregulated, while after 24 h, pro‐inflammatory cytokines and IL‐13 (an anti‐inflammatory cytokine) were downregulated and upregulated, respectively. In addition, the cytokine levels of IL‐10 were significantly increased in the cell culture supernatant after 24 h. From the traditional point of view, excretory/secretory proteins (ESP) of helminths can shift immune responses from Th1 toward Th2.^64^, ^65^, ^66^ Recently, Lin et al.^67^ showed that HCF attenuates inflammatory responses in macrophage cell lines (both mice [peritoneal macrophages and RAW 264.7 cells] and human [THP‐1] macrophages), which supports our findings. In a study by Pan et al.,^68^ the effects of ESPs derived from *E. granulosus* protoscoleces on the differentiation of CD19^+^B cells, which were isolated from the spleen of normal or infected C57BL/6 or TLR‐2−/− mice, were investigated and suggested that these ESPs can modulate immune responses via stimulating B cells to generate B10 cells with higher levels of IL‐10. In addition, they suggested that this pathway is most probably activated via Toll‐like receptor (TLR) 2. It was suggested that EVs originated from *E. granulosus* may be a cargo for ESP from different development stages of the parasite, and are employed by the parasite to orchestrate the host immune responses in its niche.^69^ In addition, the immune responses are supposed to be specifically associated with the CE type. Active CE was suggested to be correlated with Th1/Th2/regulatory responses, while inactivated CE was associated with Th1/inflammatory responses containing components of wound healing responses.^70^ HCF mostly elicit the Th2‐dependent immune responses,^71^ although there are studies indicating increased pro‐inflammatory responses in host cells treated by HCF.^72^, ^73^ In support of our findings, it was elucidated that HCF‐ derived EVs downregulated the expression of IL‐1β and IL‐17, and increased the expression of IL‐10 and TNF‐α in sheep PBMC, 24 h after exposure.^26^ In a recently published study, it was suggested that inflammation, airways resistant, and the number of eosinophils were reduced in mouse lung epithelial cells (MLE‐12) pretreated with *Aspergillus* proteins upon treatment with HCF‐derived EVs.^74^

However, although the main mechanism behind the communication between parasites‐derived EVs and their host remains unclear, it seems that the immunomodulatory effects of HCF EVs back to their contents. In addition, the contact duration between EVs and host cells has critical effects on the expression pattern of pro‐ and anti‐inflammatory cytokines. The different immune responses against CE, based on different stage and duration of contact, can increase the chance of finding a promising way not only for manipulation of immune responses by helminths, but also development of stage‐specific diagnosis approaches for CE.

## 5 CONCLUSION

In summary, EVs were successfully isolated from HCF with average size 130.6 nm. The EVs were taken up by THP‐1 after 4 h. In addition, EVs upregulated the mRNA levels of pro‐inflammatory cytokines after 3 h (IL‐1β, IL‐15, IL‐8 and IFN‐γ), while they were decreased 24 h after incubation, compared to housekeeping gene. The levels of anti‐inflammatory cytokines revealed statistically significant upregulation 24 h after co‐incubation. Taken together, lot of things about the communication between HC and host, during development of the metacestode stage, are still unclear, but it seems that HC is able to manipulate and modulate the immune responses of its host via secreted EVs.

## AUTHOR CONTRIBUTIONS

**Mojdeh Khosravi:** Investigation (equal); methodology (equal); visualization (equal); writing – original draft (equal). **Hanieh Mohammad Rahimi:** Formal analysis (equal); investigation (equal); software (equal); visualization (equal). **Abdoreza Nazari:** Formal analysis (equal); investigation (equal); validation (equal); visualization (equal). **Kavei Baghaei:** Investigation (equal); software (equal); validation (equal); writing – review and editing (equal). **Hamid Asadzadeh Aghdaei:** Project administration (equal). **Shabnam Shahrokh:** Project administration (equal); writing – review and editing (equal). **Meysam Sharifdini:** Resources (equal); validation (equal). **Ana Claudia Torrecilhas:** Methodology (equal); supervision (equal); validation (equal); writing – review and editing (equal). **Fatemeh Mehryab:** Investigation (equal); validation (equal); visualization (equal). **Hamed Mirjalali:** Conceptualization (lead); formal analysis (equal); supervision (lead); writing – original draft (lead). **Faezeh Shekari:** Conceptualization (equal); methodology (equal); validation (equal). **Mohammad Reza Zali:** Project administration (equal); resources (equal); writing – review and editing (equal).

## FUNDING INFORMATION

This study was financially supported by the Research Institute for Gastroenterology and Liver Diseases, Shahid Beheshti University of Medical Sciences with grant number: RIGLD‐1029 and Royan institute with grant number: 97000121.

## CONFLICT OF INTEREST STATEMENT

The authors declare that they have no conflict of interest.

## CONSENT FOR PUBLICATION

All authors declare that they have seen and approved the submitted version of this manuscript.

## Supporting information


Figure S1.

Figure S2.

Figure S3.

Figure S4.

Figure S5.

## Data Availability

All generated data from the current study are included in the article and its supplementary materials and data.
