# Supplementary figures and images for: Characterisation of extracellular vesicles isolated from hydatid cyst fluid and evaluation of immunomodulatory effects on human monocytes
Source: J Cell Mol Med. 2023 Aug 2;27(17):2614–25. doi: 10.1111/jcmm.17894 (PMC10468670; doi:10.1111/jcmm.17894)

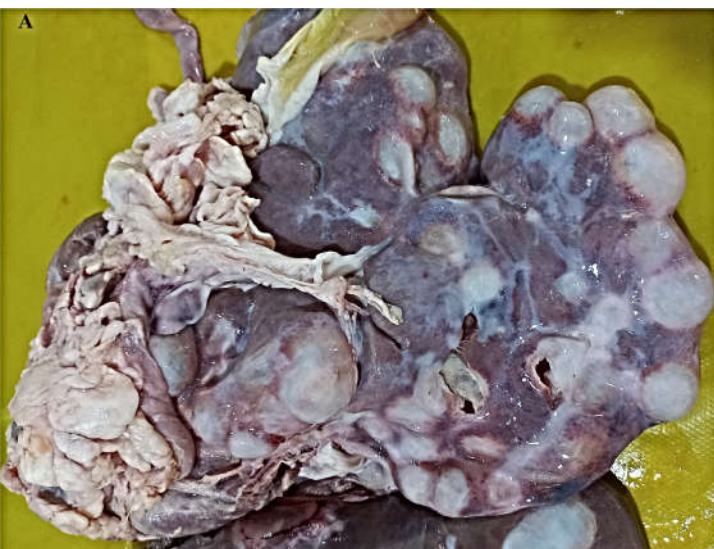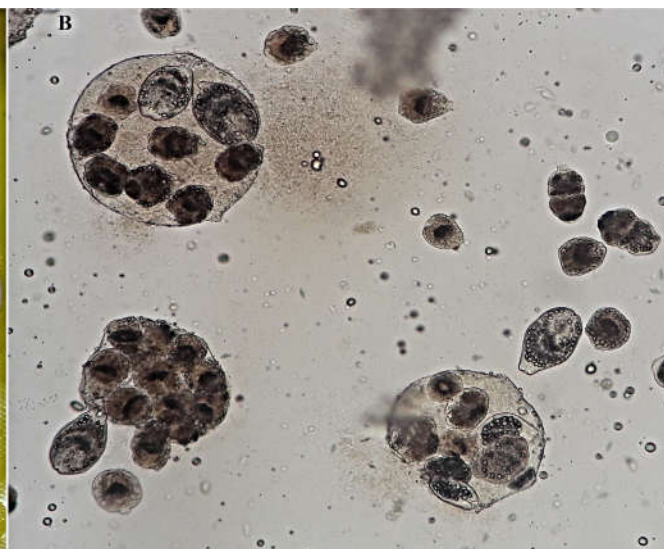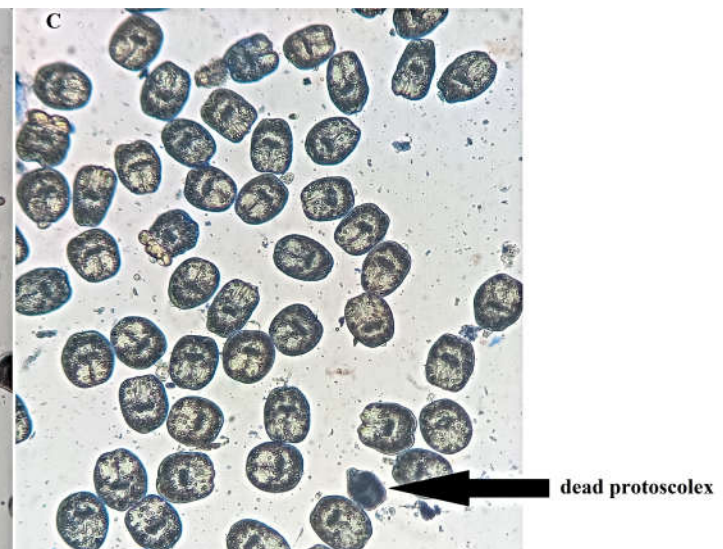

Supplement: Supplementary file 1 — Figure S1. [file JCMM-27-2614-s005.pdf]

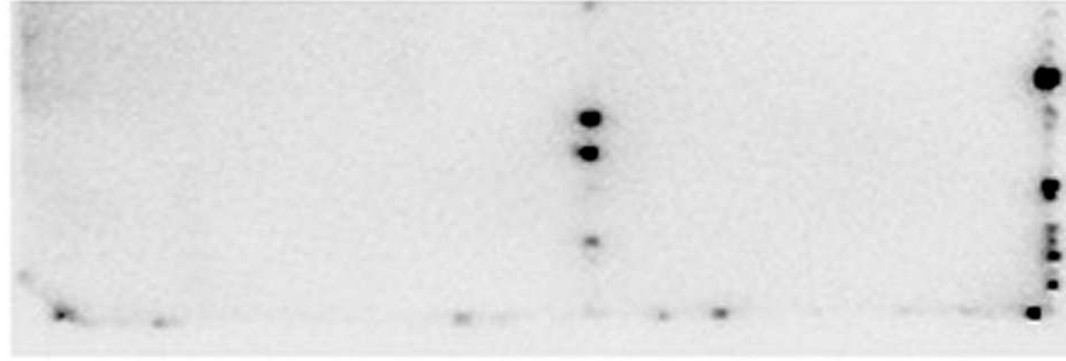

Calnexin

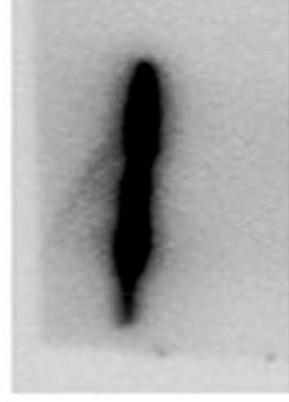

Positive control  
for calnexin

Supplement: Supplementary file 2 — Figure S2. [file JCMM-27-2614-s002.pdf]

Molecular  
weight marker

20  $\mu$ g EV protein

10  $\mu$ g EV protein

5  $\mu$ g EV protein

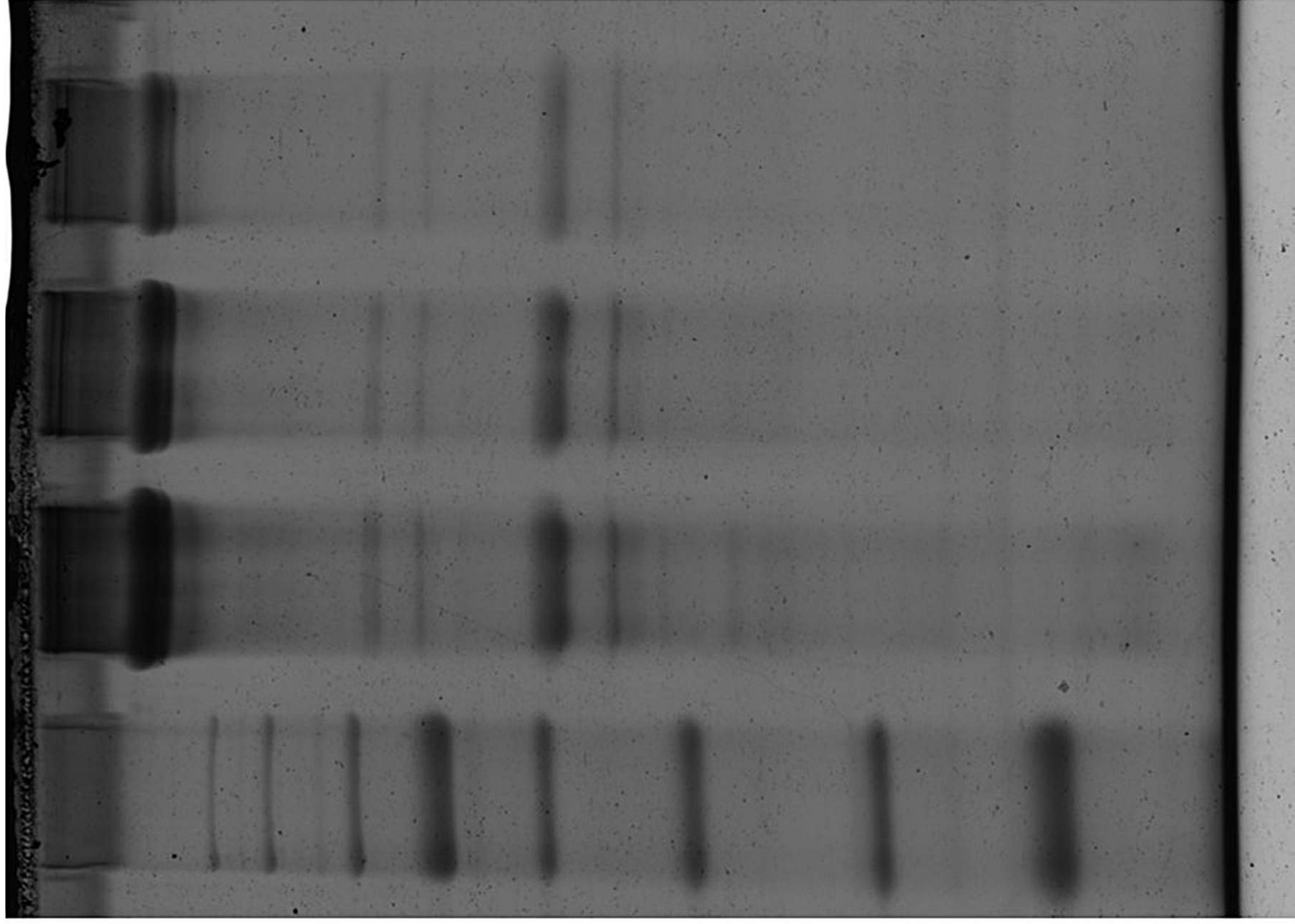

Supplement: Supplementary file 3 — Figure S3. [file JCMM-27-2614-s004.pdf]

**A**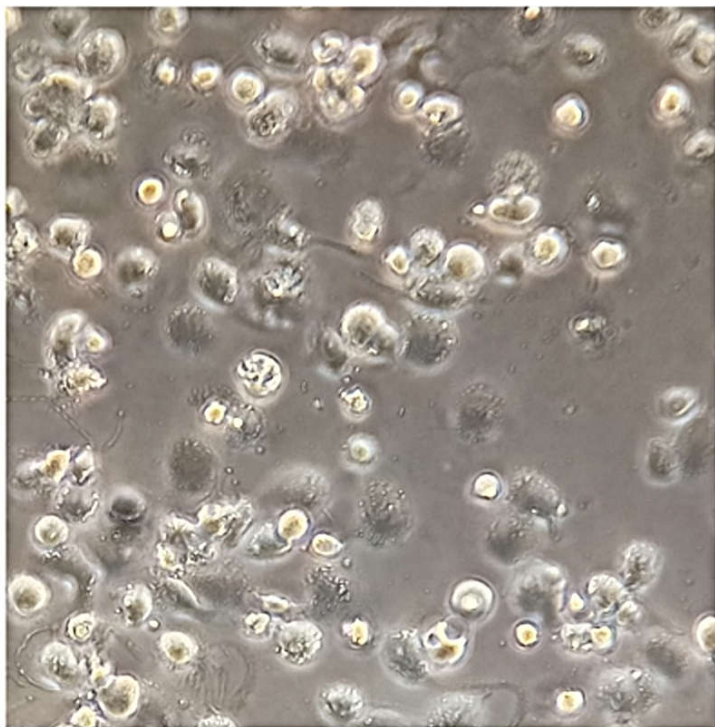**B**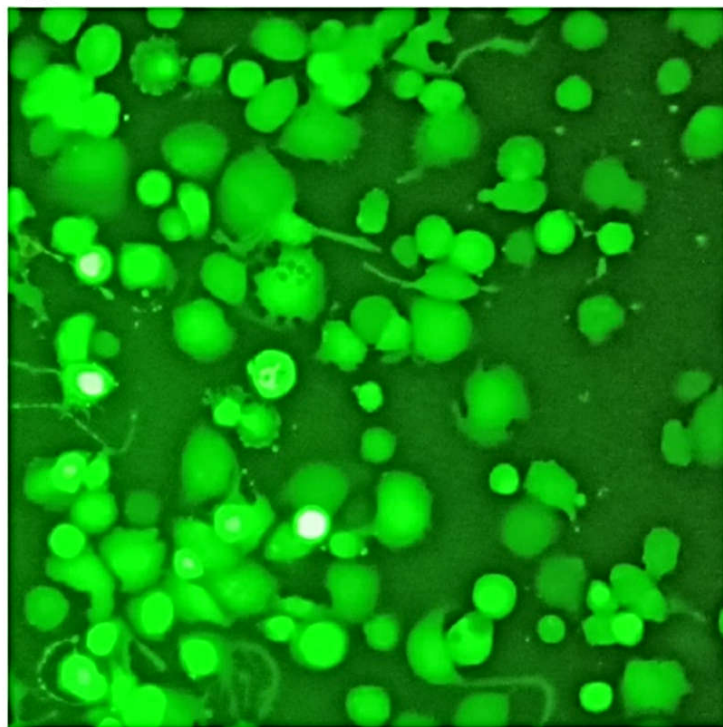**C**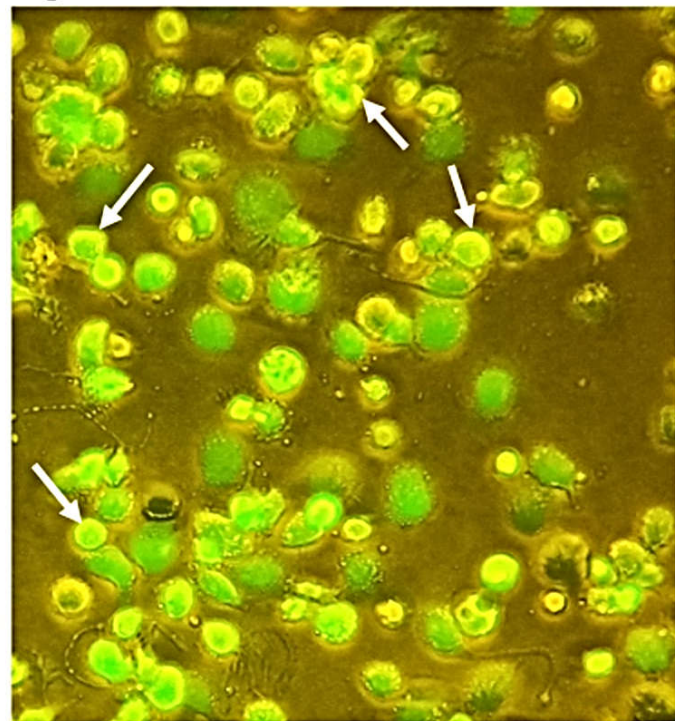

Supplement: Supplementary file 5 — Figure S5. [file JCMM-27-2614-s001.pdf]
