# Supplementary material for: Characterisation of extracellular vesicles isolated from hydatid cyst fluid and evaluation of immunomodulatory effects on human monocytes
Source: J Cell Mol Med. 2023 Aug 2;27(17):2614–25. doi: 10.1111/jcmm.17894 (PMC10468670; doi:10.1111/jcmm.17894)
Supplement: Supplementary file 4 — Figure S4. [file JCMM-27-2614-s003.pdf]

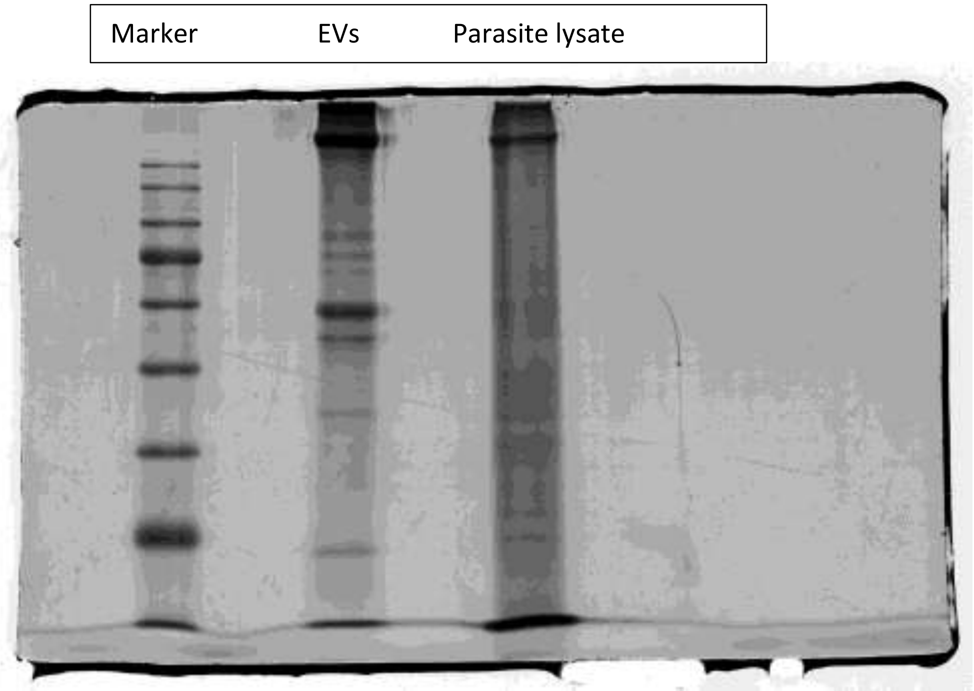

A) SDS-PAGE of 15 µg extracted protein from **1)** EVs and **2)** parasite lysate

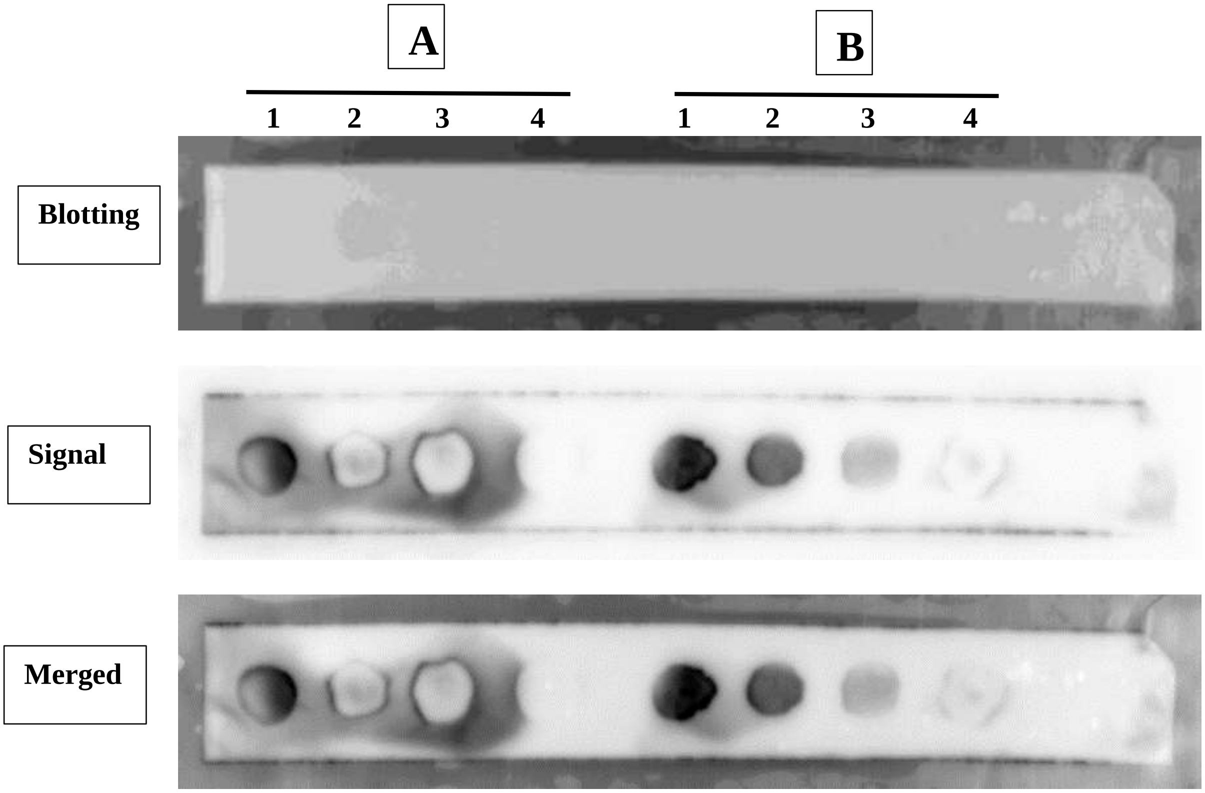

B) Dot blot of extracted proteins from **1)** EV and **2)** parasite lysate with different concentrations: **a)** 5 µg, **b)** 1 µg, **c)** 0.5 µg, **d)** 0.1 µg
